# Functional analysis of metagenomes and metatranscriptomes using SEED and KEGG

**DOI:** 10.1186/1471-2105-12-S1-S21

**Published:** 2011-02-15

**Authors:** Suparna Mitra, Paul Rupek, Daniel C Richter, Tim Urich, Jack A Gilbert, Folker Meyer, Andreas Wilke, Daniel H Huson

**Affiliations:** 1 Center for Bioinformatics ZBIT, Tübingen University, Sand 14, 72076 Tübingen, Germany; 2 Eurofins Medigenomix GmbH, Anzinger Str. 7, 85560 Ebersberg, Germany; 3 Department of Genetics in Ecology, Vienna Ecology Center, University of Vienna, 1090 Vienna, Austria; 4 Centre for Geobiology, University of Bergen, 5007 Bergen, Norway; 5 Department of Ecology and Evolution, The University of Chicago, Chicago, IL 60637, USA; 6 Mathematics and Computer Science Division, Argonne National Laboratory, Argonne, IL 60439, USA

## Abstract

**Background:**

Metagenomics is the study of microbial organisms using sequencing applied directly to environmental samples. Technological advances in next-generation sequencing methods are fueling a rapid increase in the number and scope of metagenome projects. While metagenomics provides information on the gene content, metatranscriptomics aims at understanding gene expression patterns in microbial communities. The initial computational analysis of a metagenome or metatranscriptome addresses three questions: (1) Who is out there? (2) What are they doing? and (3) How do different datasets compare? There is a need for new computational tools to answer these questions. In 2007, the program MEGAN (MEtaGenome ANalyzer) was released, as a standalone interactive tool for analyzing the taxonomic content of a single metagenome dataset. The program has subsequently been extended to support comparative analyses of multiple datasets.

**Results:**

The focus of this paper is to report on new features of MEGAN that allow the functional analysis of multiple metagenomes (and metatranscriptomes) based on the SEED hierarchy and KEGG pathways. We have compared our results with the MG-RAST service for different datasets.

**Conclusions:**

The MEGAN program now allows the interactive analysis and comparison of the taxonomical and functional content of multiple datasets. As a stand-alone tool, MEGAN provides an alternative to web portals for scientists that have concerns about uploading their unpublished data to a website.

## Background

Metagenomics seeks to understand microbial communities by DNA sequencing. Deeper sequencing and better reference databases are advancing the potential and success of such analyses. While metagenomics provides information on the gene content of a microbial community, metatranscriptomics promises to reveal the actual metabolic activities of this community at a specific time and place, and how those activities change in response to environmental forces or biotic interactions.

A number of different systems and resources for metagenome or similar analysis, which are offered in the form of databases, web portals, web services and basic stand-alone programs [1-11]. These resources are mainly focused on the analysis of individual metagenomes and currently do not have the capacity for rapid and highly interactive comparison of multiple datasets. Furthermore, many of these resources are suitable only for taxonomic analysis. In our experience, only the MG-RAST web server [1,11] currently provides a readily useable service for analyzing a new metagenomic dataset. However, while web portals are attractive because they offer large computational resources for data analysis, some scientists have concerns about uploading their unpublished data to a website. To address this problem of taxonomic analysis, the program MEGAN [12] was published in 2007, as the first stand-alone interactive tool for analyzing the taxonomic content of a dataset. A subsequent version of the program was developed that allows one to compare the taxonomic content of different datasets [13,14]. MEGAN is easy to install and use, and requires only a BLAST output file as input to operate. The program is designed to allow both high-level analysis that summarizes data at different ranks of the NCBI taxonomy, and detailed analysis that drills down to individual reads and their BLAST matches. The goal of this paper is to describe new features of MEGAN that allow the functional analysis of a microbial community. This type of analysis can assist in understanding biochemical processes or in estimating the influence of environmental changes on biospheres. The next major release of MEGAN allows the functional analysis of metagenomic and metatranscriptomic datasets using the SEED classification, based on the given BLAST file. For comparative purposes, one can simultaneously map multiple datasets onto the SEED hierarchy and also compute distance matrices on datasets based on their SEED content.

For the pathway analysis, the field of systems biology already possesses a high-quality database, namely the Kyoto Encyclopedia for Genes and Genomes (KEGG) [15]. MEGAN provides a KEGG analysis window that reports which KEGG pathways are present in a dataset and allows one to then inspect these pathways; for example, MEGAN can capture all reads that are mapped to a given pathway of interest.

## Results and discussion

### SEED analysis with MEGAN

MEGAN performs a taxonomic analysis of a dataset by mapping reads onto different taxa in the NCBI taxonomy, depending on the phylogenetic footprint of the gene that a read contains (using the lowest common ancesstor algorithm, as described in [12]). The result is displayed as a rooted tree where the nodes represent the different taxa and are scaled and labeled by the number of reads assigned to the taxon.

Now, as a new feature, MEGAN uses the SEED classification [1] for functional analysis. In this classification, genes are assigned to functional roles and different functional roles are grouped into subsystems. The SEED classification can be represented by a rooted tree where the internal nodes represent the different subsystems and where the leaves represent the functional roles. Note that the tree is “multi-labeled” in the sense that different leaves may represent the same functional role, if a role occurs in different subsystems. The current SEED tree has about 10,000 nodes.

**Figure 1:**
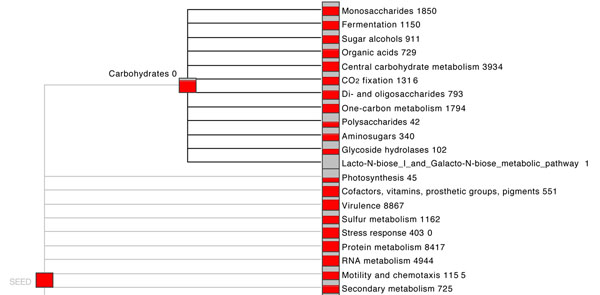
**SEED-based functional assignment.** Part of a SEED-based functional analysis of a marine metagenome sample. Each item represents a functional role in the SEED and is labeled by the number of reads assigned to this.

To perform a functional analysis, MEGAN assigns each read to the functional role of the highest scoring gene in a BLAST comparison against a protein database. Figure 1 shows a part of the functional analysis of a marine metagenome sample. The program reports the numbers of reads assigned to each functional role.

### KEGG analysis with MEGAN

To perform a KEGG analysis, MEGAN attempts to match each read to a KEGG orthology (KO) accession number, using the best hit to a reference sequence for which a KO accession number is known. MEGAN then calculates the number of hits to each KEGG pathway and reports these numbers to the user. The user can request to see the hits to a given pathway and an appropriate image of the pathway is generated by coloring the pathways based on the KEGG mapping. MEGAN allows one to analyze several datasets together, using different colors to show which parts of a pathway are present in which datasets. Because different genes that are present in different organisms in a consortium of microbes will often not operate together in a single pathway, MEGAN allows one to restrict the pathway analysis to a set of one or more taxa in the NCBI taxonomy [15].

### Comparing functional content of different datasets

**Figure 2:**
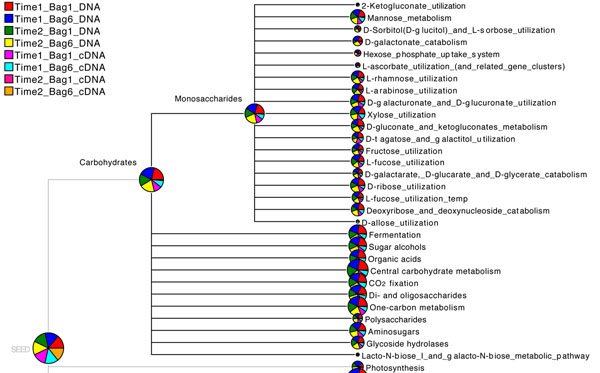
**SEED-based functional comparison.** Part of a SEED-based functional comparison of eight marine samples.

**Figure 3:**
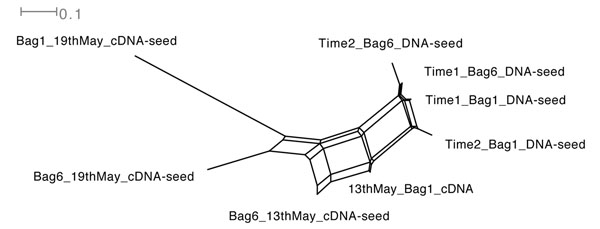
**Network comparison based on functional content**. Network comparison of eight marine samples based on functional content.

**Figure 4:**
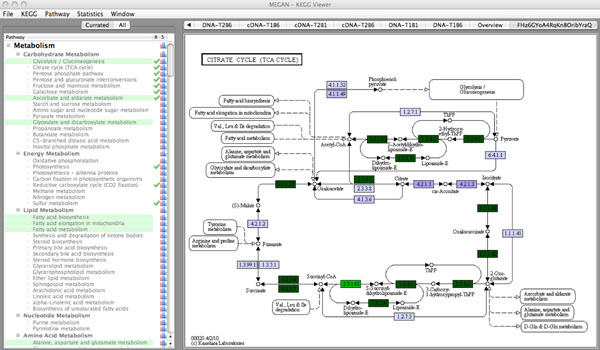
**MEGAN’s KEGGviewer.** MEGAN’s KEGGviewer, showing a comparison of the ‘citrate cycle’ for a metagenome sample (blue) and an associated transcriptome sample (green)

MEGAN supports the simultaneous analysis and comparison of the taxonomic content and now also the functional content of multiple datasets in several ways. The functional content of a set of metagenomes can be simulteniously opened and compared using a new SEED-based tree view (see Figure 2). Furthermore a collection of datasets can be compared using six different ecological indices, the UniFrac measure [2] and different distance analysis techniques (see [14] for details). As an example Figure 3, shows the comparison of eight Bergen marine samples based on their functional content using Goodall’s index. Finally it is also possible to compare pathways present in multiple microbial communities using MEGAN’s KEGGviewer. One can compare multiple datasets using different colors see Figure 4.

### MEGAN and MG-RAST

**Figure 5:**
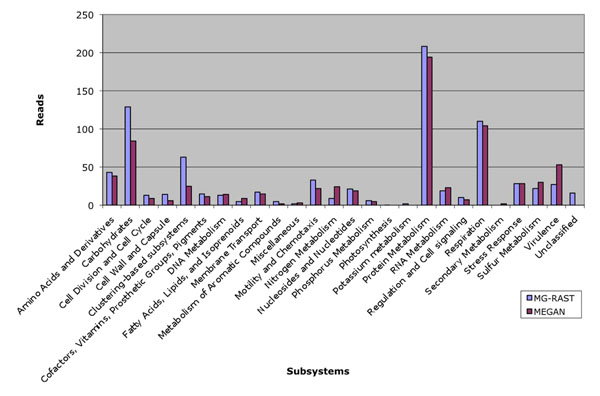
**Comparison of MEGAN and MG-RAST assignments using a small unpublished dataset.** Absolute comparison of MEGAN and MG-RAST assignments for a small unpublished dataset of 1408 sequence reads.

A first comparison between MEGAN and MG-RAST was performed with a small subset of an FLX-titanium pyrosequencing dataset (Roche-454) obtained from a hydrothermal vent microbial community. Out of a total of 1408 sequences, MG-RAST assigned 831 functions and MEGAN 727; the latter amounts to 88% of the assignments from MG-RAST. Figure 5 shows the comparison of assignments on the highest SEED subsystem hierarchy. Both tools assign a very similar number of sequences to most of the subsystems, although some subsystems vary by a factor of 2 or more.

**Figure 6:**
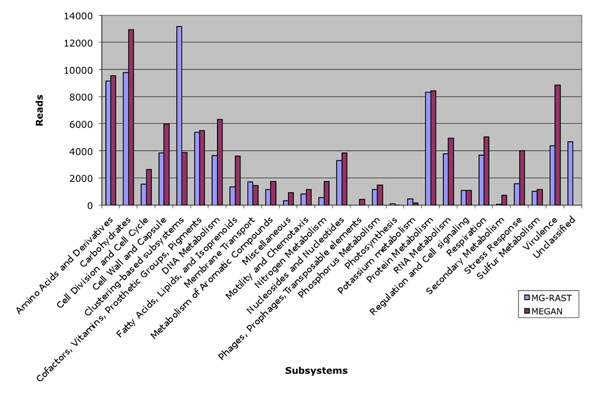
**Comparison of MEGAN and MG-RAST assignments using a published marine sample.** Absolute comparison of MEGAN and MG-RAST assignments for a published marine sample (Time1-Bag1-DNA sample from the Bergen marine datasets) of 209, 073 sequence reads.

In addition, we have tested our program on a published dataset (the Time1-Bag1-DNA sample from the Bergen marine datasets, see [16] for details). Out of a total of 209, 073 sequences MG-RAST assigned 86, 167 functions and MEGAN 97, 748; here MEGAN has more assignments. Figure 6 shows the comparison of MEGAN and MG-RAST assignments for these data. In both cases, we see that the number of reads assigned to different subsystems by MG-RAST and MEGAN are very similar, but with some large differences. These differences are to be expected, as MEGAN and MG-RAST use different reference databases for their analysis. In the MEGAN analysis, we used the March’10 version of the NCBI-NR (non-redundant) protein database [17], whereas MG-RAST (version 2) used an expert-annotated, NR database build from all organisms curated in the SEED.

#### For pathway analyses using KEGG metabolic maps

We have performed pathway analyses for pooled DNA samples from the PLM-Bergen datasets. To compare MEGAN against MG-RAST, we have concentrated on two different pathways, namely the ‘citrate cycle (TCA cycle)’ and ‘photosynthesis’.

**Figure 7:**
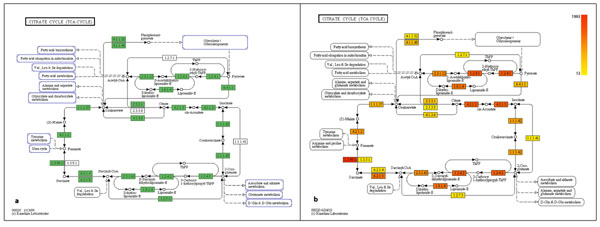
**Comparison of MEGAN and MG-RAST in the ‘citrate cycle KEGG map’.** Figure shows the ‘citrate cycle KEGG map’ obtained using (a) MG-RAST web server and (b) MEGAN’s KEGGviewer, for four pooled DNA samples from PLM-Bergen datasets, based on SEED subsystems.

The citrate cycle (TCA cycle): The citric acid cycle is of central importance for cells that use oxygen as part of cellular respiration. We compared the results for this cycle produced by both tools (MEGAN and MG-RAST) using pooled DNA samples from the PLM-Bergen datasets. The resulting pathway graphs (Figure 7) look very similar in both the cases when we consider only the presence or absence of enzymes. MG-RAST only colors the enzyme nodes if they are present (labelled in green in Figure 7a) in the pathways, but MEGAN’s KEGGviewer is able to scale the color of the enzymes according to their read abundances (scaled in yellow to red in Figure 7b). This color gradient can help in understanding the enzyme kinetics, as the abundance of reads assigned to an enzyme can be proportional to the turnover frequency (TOF) associated with that enzyme. If we assume that the TCA cycle is the most prominent cycle of a cell, then all the enzymes are supposed to be present with some level of abundance. Here, we can see some nodes (EC 1.2.7.1, EC 2.3.3.8, EC 1.1.1.41, EC 1.3.5.1) are absent in the MG-RAST analysis, but are present with a very low read abundance in MEGAN’s KEGGviewer.

**Figure 8:**
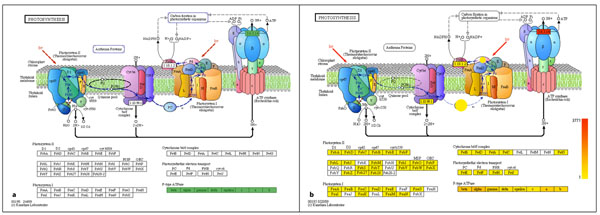
**Comparison of MEGAN and MG-RAST in the ‘photosynthesis KEGG map’**. Figure shows the ‘photosynthesis KEGG map’ obtained using (a) MEG-RAST web server and (b) MEGAN’s KEGGviewer, for four pooled DNA samples from PLM-Bergen datasets, based on SEED subsystems.

Photosynthesis: For all the datasets considered, for the photosynthesis KEGG map, MG-RAST only provides hits associated with ‘F-type ATPase’, ‘Cytochrome b6/f complex (PetC)’ and ‘Photosynthetic electron transport (PetH)’. For example, using the four pooled DNA samples from the PLM-Bergen datasets, the MG-RAST server states that all the ‘F-type ATPase’ enzymes are present (labeled in green in Figure 8a). But with MEGAN’s KEGGviewer we can also see the Photosystem I or II, ‘Cytochrome b6/f complex (PetC)’ and reads associated with ‘Photosynthetic electron transport (PetH) (scaled in yellow to red in Figure 8b).

These results show the advantages of MEGAN’s KEGGviewer for KEGG-based functional analysis.

## Conclusions

Here we have presented the functional assignment module of MEGAN. Using two examples, we show that MEGAN and SEED obtain comparable results to MG-RAST. With this new version of MEGAN, researchers can perform a functional analysis using the SEED classification. Because MEGAN performs this analysis directly from the BLAST input file, no additional calculations are required. Thus, MEGAN provides a stand-alone alternative to the MG-RAST server. In future work, we plan to integrate MEGAN into the MG-RAST portal. This will bring together the highly interactive features of MEGAN and the computational power of MG-RAST together.

## Methods

### Data preparation

For the first case study, we used a small subset consisting of 1408 sequences from an FLX-titanium pyrosequencing dataset (Roche-454) obtained from a hydrothermal vent microbial community. We used eight marine datasets from Plymouth Marine Laboratory, consisting of four metagenomes (DNA) and four metatranscriptomes (cDNA) from a mesocosm experiment performed in Bergen, Norway (see [16] for details). In this paper, we refer to these as the PLM-Bergen datasets. All metagenomes and metatranscriptomes were aligned against the NCBI-NR database using the BLASTX tool [18]. The results were then imported into MEGAN [12] (with default parameters), using the ‘Import from BLAST’ option and saved as MEGAN own ‘rma files’.

### Functional Assignment Based on MEGAN-SEED

MEGAN places each read of a given dataset onto one of the taxa (or “nodes”) of the NCBI taxonomy, based on the BLAST matches provided for the read, using the LCA algorithm. For functional assignment in a similar fashion, MEGAN provides a hierarchical representation using the SEED classification. Normally, reads are mapped to the NCBI taxonomy, and the program provides the exact numbers of reads assigned to any given node and the number of hits to any nodes in the subtree rooted at the node. In a similar fashion for the functional analysis, reads are mapped to SEED subsystems using the ‘seed2ncbi.gz’ file from the SEED server.

### Multiple metagenome comparison using the functional content

#### Multiple Comparison Tree-view

After opening all the samples in MEGAN, we compared the taxonomic content using the ‘Compare’ menu item to obtain the comparison in a new window. Choosing the ‘SEED’ menu from the comparison window allowed us to get a functional comparison of the samples directly in a new window (Figure 2).

#### Multiple Comparison Network-view

To compare six PLM-Bergen marine samples, based on their functional content, we chose ‘Networks’ from the ‘Option’ menu of MEGAN’s SEEDviewer to see the network comparison view of multiple datasets that are under consideration (Figure 3). Moreover, one has six different choices of distance measures to compute the networks (see [14] for details).

### Comparison of MEGAN and MG-RAST assignments

MG-RAST is a leading service for functional annotation. To test the functional assignment of MEGAN, we compared MEGAN’s functional assignment with MG-RAST’s assignment in two datasets which are described using two case studies below.

We first performed MEGAN-SEED annotations on an unpublished dataset of 1408 sequences (454 FLX-titanium pyrosequencing) of a metatranscriptome obtained from a hydrothermal vent microbial community and compared them with the parallel assignments obtained by MG-RAST. We then performed the same study with a published marine dataset (the Time1-Bag1-DNA sample from the Bergen datasets; 209, 073 sequences) and compared the results with MG-RAST’s assignment.

### Pathway analyses based on KEGG

MEGAN includes a module called ‘KEGGviewer’ for the analysis of metagenomic data in the context of pathways. It is designed to consume a list of RefSeq accession numbers and maps them to KEGG orthologies (KO numbers). The functionality of this program has been compared to MG-RAST, which also provides basic pathway analysis methods for some of the above mentioned PLM-Bergen marine samples by selecting different metabolic pathways.

## Authors' contributions

SM and DHH designed the project and wrote the manuscript. PR performed the KEGG analysis and wrote necessary codes. DHH wrote necessary codes for SEED. SM performed all the BLAST, SEED analysis, and multiple comparison. All other authors contributed in MG-RAST analysis and in helpful discussion.

## Competing interests

The authors declare that they have no competing interests.
